# Supplementary material for: Impact of surface and pore characteristics on fatigue life of laser powder bed fusion Ti–6Al–4V alloy described by neural network models
Source: Sci Rep. 2021 Oct 14;11:20424. doi: 10.1038/s41598-021-99959-6 (PMC8516886; doi:10.1038/s41598-021-99959-6)
Supplement: Supplementary file 1 — Supplementary Information. [file 41598_2021_99959_MOESM1_ESM.docx]

**Supplementary material**

**Impact of Surface and Pore Characteristics on Fatigue Life of Laser Powder Bed Fusion Ti-6Al-4V Alloy Described by Neural Network Models**

Seunghyun Moon^1^, Ruimin Ma^1^, Ross Attardo^2^, Charles Tomonto^2^, Mark Nordin^3^, Paul Wheelock^3^, Michael Glavicic^3^, Maxwell Layman^3^, Richard Billo^4^, Tengfei Luo^1,5,*^

1. Department of Aerospace and Mechanical Engineering, University of Notre Dame, IN 46556

2. 3D Printing Center, Johnson & Johnson, Miami, FL 33126

3. Rolls-Royce Corporation, 450 S. Meridian St., Indianapolis, IN 46225

4. Department of Computer Science and Engineering, University of Notre Dame, IN 46556

5. Department of Chemical and Biomolecular Engineering, University of Notre Dame, IN 46556

* corresponding author: tluo@nd.edu


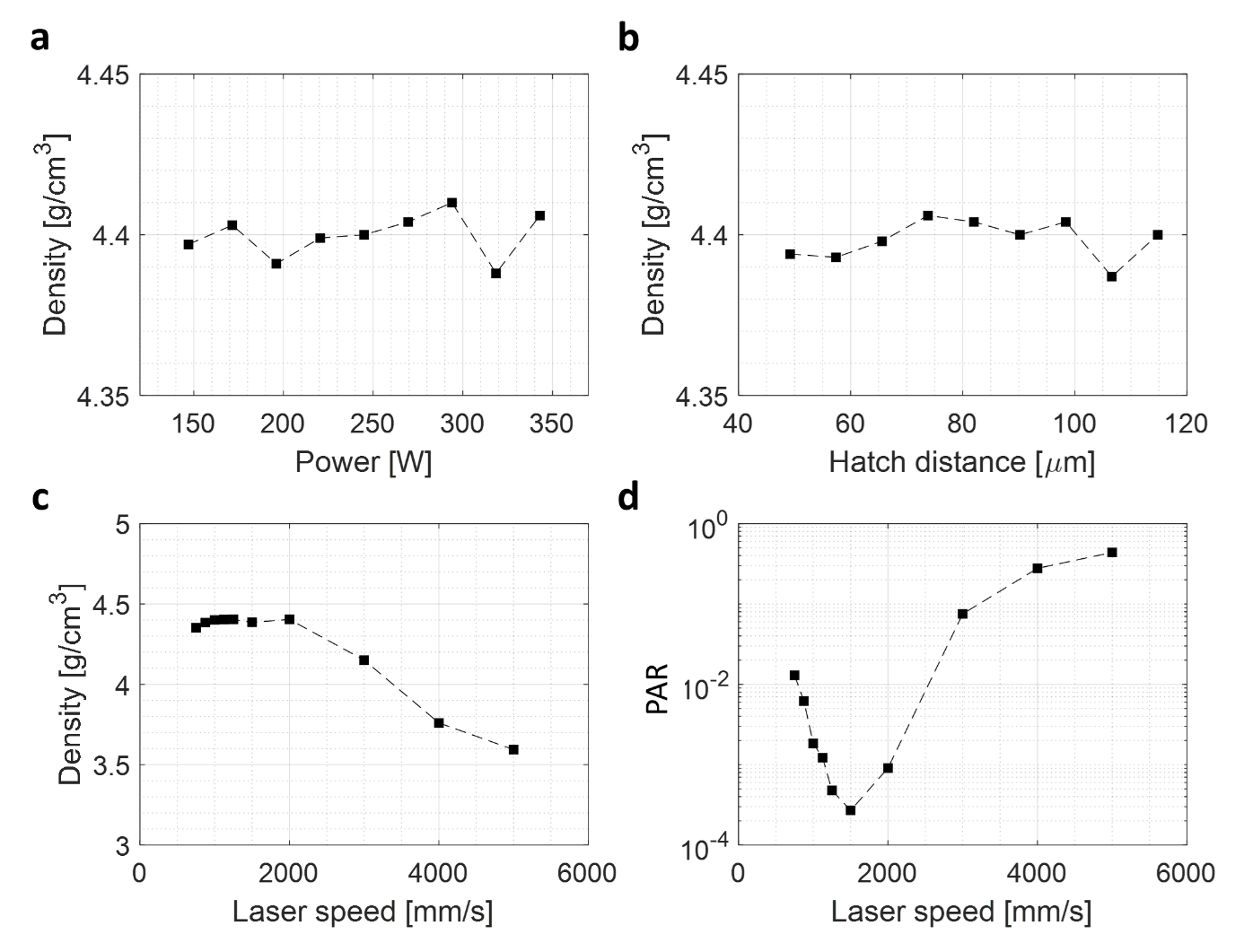


Figure S1. Preliminary investigation for LPBF printing parameters. After printing was complete, the build plate was removed from the machine, and samples were removed by prying them off the support with a screwdriver. (a) The density was measured through Archimedes’ principle. The power range was from 147 to 343 W. (b) Plot of hatch distance vs. density. Hatch distance was varied from 49.2 to 114.8 µm. (c) Density variations depending on laser speed, which varied from 750 to 2000 mm/s. (d) Variation of PAR depending on laser speed. PAR values were estimated from mirror-polished surfaces.


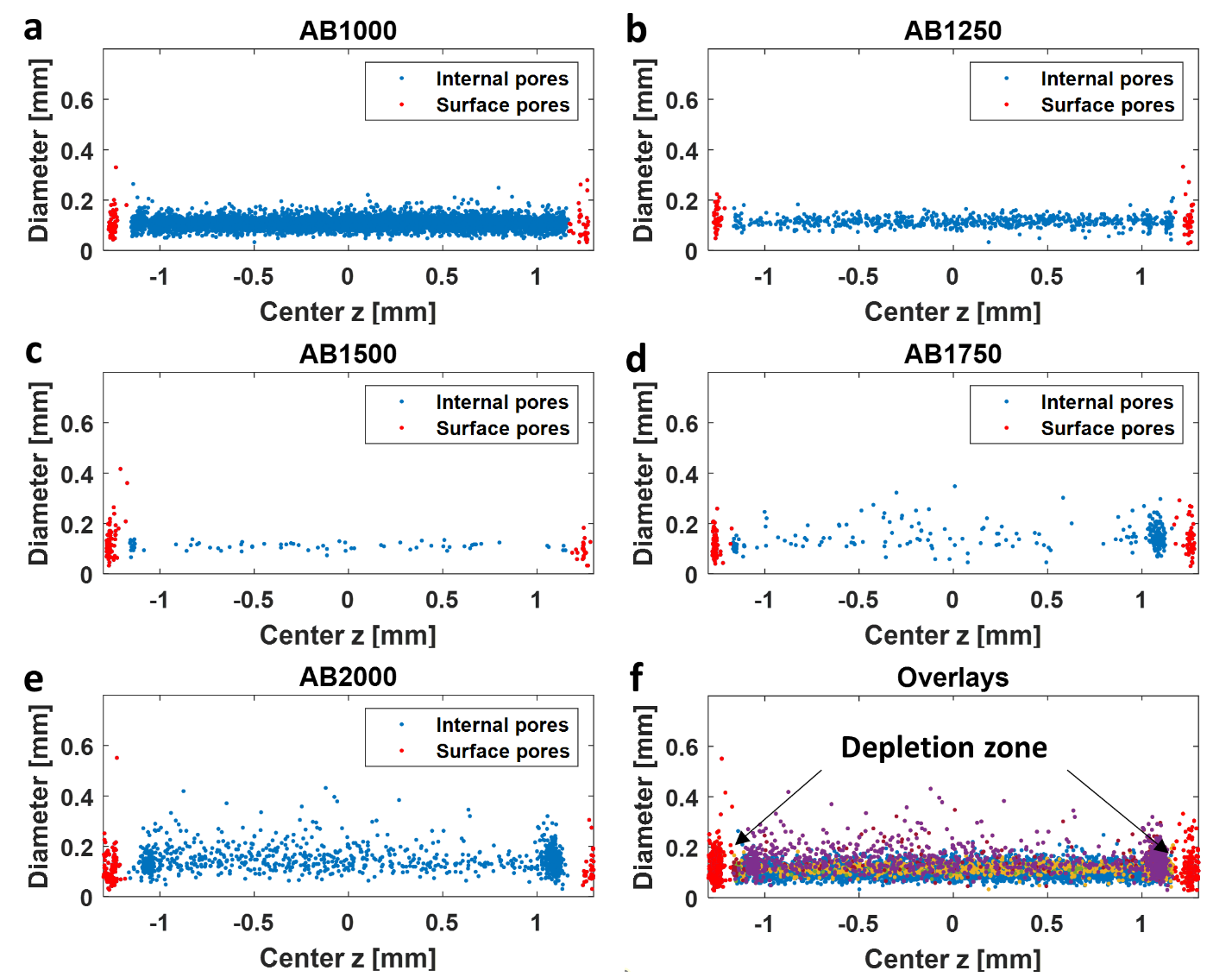


Figure S2. Depletion zone created by the contour scans with hatch speed 1000 mm/s (a), 1250 mm/s (b), 1500 mm/s (c), 1750 mm/s (d), 2000 mm/s (e) and overlays of all AB samples (f).


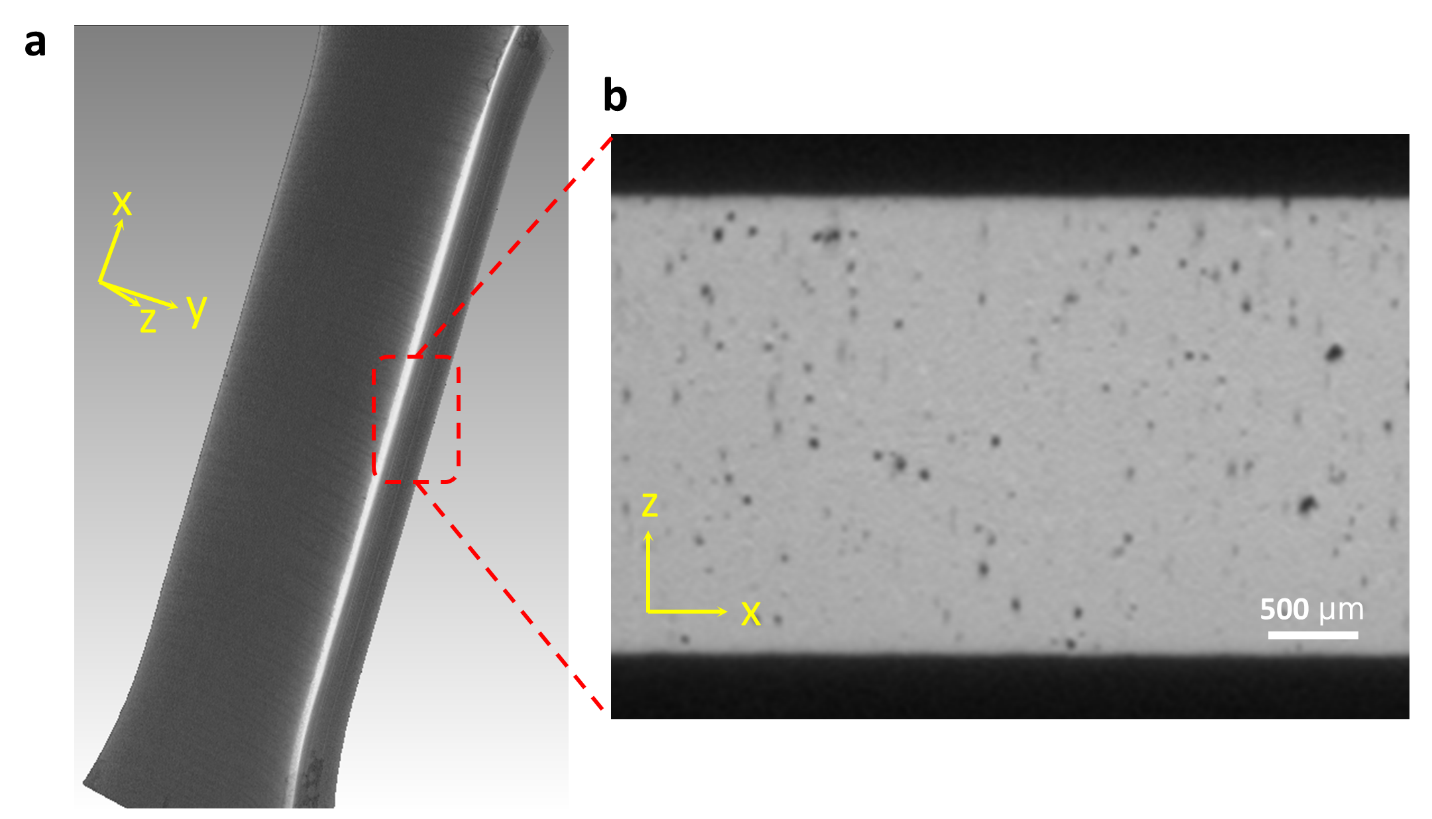


**Figure S3. (a)** Representative reconstruction of a micro-CT scanned M sample. Laser scanning speed is 750 mm/s. **(b)** Side view of the M0750 sample. Note the build direction is along the x-direction.


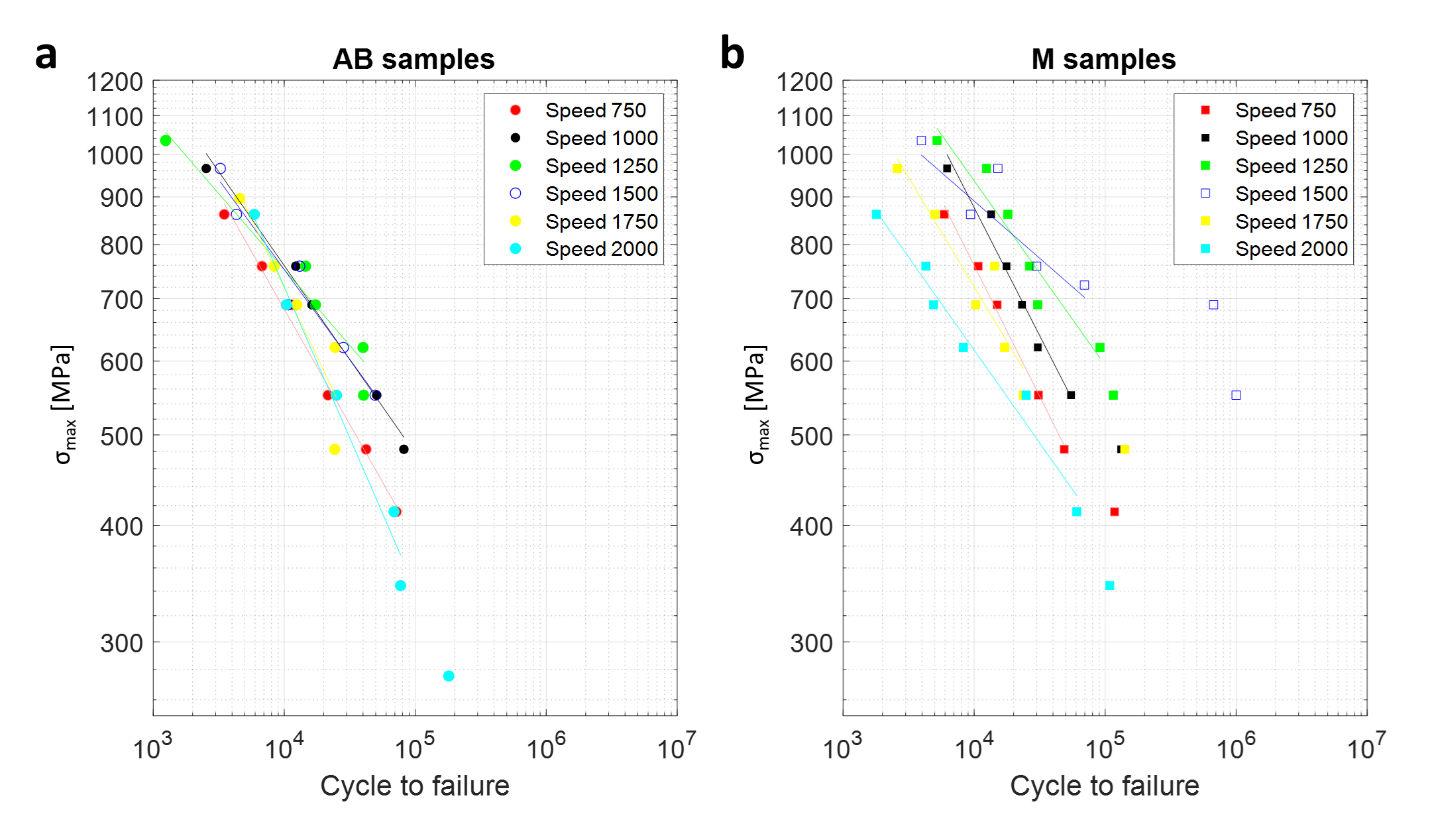


Figure S4. (a) The S-N plot for AB samples with the log-log coordination. (b) The S-N plot for M samples with the log-log coordination. The fitting results included only HCF region of data.

**Table S1.** Fitting results for linear-log form and Basquin’s law.

| Laser speed | Surface finish | Linear-log | | | Basquin’s law | | |
| --- | --- | --- | --- | --- | --- | --- | --- |
|  |  | *a* | *b* | *R^2^* | *m* | *c* | *R^2^* |
| 750 | AB | -150.932 | 2088.53 | 0.991 | -0.24701 | 6636.048 | 0.991 |
| 1000 |  | -140.342 | 2069.577 | 0.997 | -0.20155 | 4861.745 | 0.988 |
| 1250 |  | -128.901 | 1959.846 | 0.973 | -0.16272 | 3367.228 | 0.942 |
| 1500 |  | -141.605 | 2069.427 | 0.935 | -0.19249 | 4428.899 | 0.949 |
| 1750 |  | -201.908 | 2592.175 | 0.911 | -0.2946 | 10840.79 | 0.867 |
| 2000 |  | -182.27 | 2411.629 | 0.978 | -0.32346 | 14176.66 | 0.977 |
| 750 | M | -182.842 | 2450.104 | 0.999 | -0.28041 | 10052.25 | 0.995 |
| 1000 |  | -203.719 | 2755.162 | 0.969 | -0.27459 | 10992.25 | 0.967 |
| 1250 |  | -160.508 | 2419.191 | 0.915 | -0.19788 | 5794.443 | 0.920 |
| 1500 |  | -105.702 | 1872.312 | 0.918 | -0.12257 | 2754.087 | 0.941 |
| 1750 |  | -176.618 | 2360.288 | 0.910 | -0.23456 | 6251.673 | 0.882 |
| 2000 |  | -121.23 | 1750.582 | 0.968 | -0.19913 | 3858.992 | 0.970 |


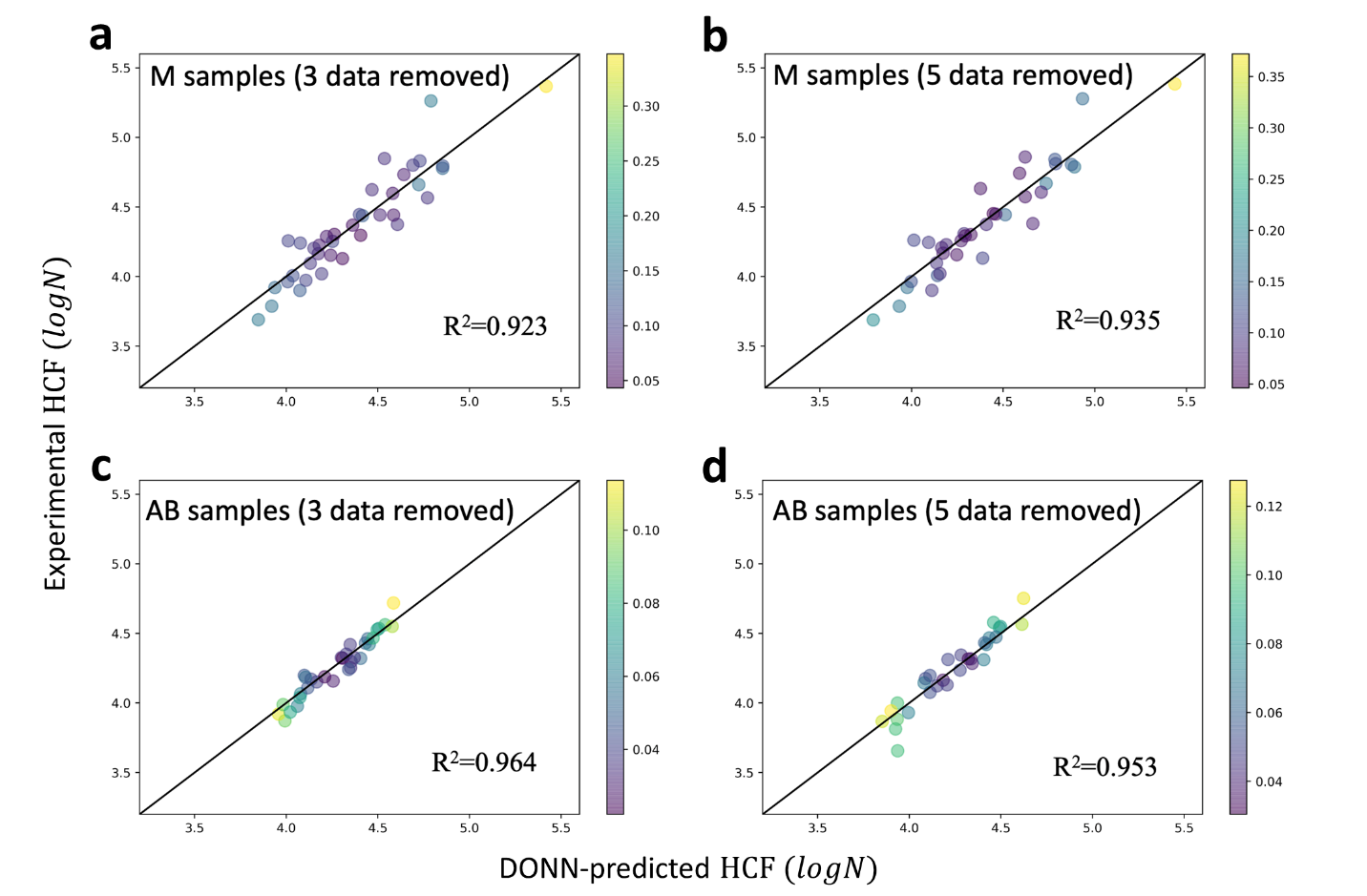


**Figure S5**. DONN prediction vs. ground truth for tests with 3 or 5 less data points for M and AB samples. In these tests, 3 or 5 data points are randomly removed from the database and the DONN is retrained using the same leave-one-out scheme.
